# Quality improvement initiatives for hospitalised small and sick newborns in low- and middle-income countries: a systematic review

**DOI:** 10.1186/s13012-018-0712-2

**Published:** 2018-01-25

**Authors:** Nabila Zaka, Emma C. Alexander, Logan Manikam, Irena C. F. Norman, Melika Akhbari, Sarah Moxon, Pavani Kalluri Ram, Georgina Murphy, Mike English, Susan Niermeyer, Luwei Pearson

**Affiliations:** 1 UNICEF New York, UNICEF House, 3 United Nations Plaza, New York, NY 10017 USA; 2 King’s College London GKT School of Medical Education, Guy’s Campus, London, SE1 1UL UK; 3 UCL Institute Epidemiology & Healthcare, 1 - 19 Torrington Place, London, WC1E 6BT UK; 4 Maternal, Adolescent, Reproductive and Child Health (MARCH) Centre and Department of Infectious Disease Epidemiology, London School of Hygiene and Tropical Medicine, Keppel Street, London, WC1E 7HT UK; 5 Department of Epidemiology and Environmental Health, 237 Farber Hall, Buffalo, NY 14214-8001 USA; 6 Office of Maternal and Child Health and Nutrition, USAID, Washington DC, USA; 7 Centre for Tropical Medicine and Global Health, Nuffield Department of Medicine Research Building, University of Oxford, Old Road Campus, Roosevelt Drive, Headington, Oxford, OX3 7FZ UK; 8 Section of Neonatology, University of Colorado School of Medicine, Aurora, CO 80045 USA

**Keywords:** Newborns, Quality improvement, Low- and middle-income countries, Systematic review, Preterm, Neonatal mortality rates, Infection control, Hospital stay

## Abstract

**Background:**

An estimated 2.6 million newborns died in 2016; over 98.5% of deaths occurred in low- and middle-income countries (LMICs). Neonates born preterm and small for gestational age are particularly at risk given the high incidence of infectious complications, cardiopulmonary, and neurodevelopmental disorders in this group. Quality improvement (QI) initiatives can reduce the burden of mortality and morbidity for hospitalised newborns in these settings. We undertook a systematic review to synthesise evidence from LMICs on QI approaches used, outcome measures employed to estimate effects, and the nature of implementation challenges.

**Methods:**

We searched Medline, EMBASE, WHO Global Health Library, Cochrane Library, WHO ICTRP, and ClinicalTrials.gov and scanned the references of identified studies and systematic reviews. Searches covered January 2000 until April 2017. Search terms were “quality improvement”, “newborns”, “hospitalised”, and their derivatives. Studies were excluded if they took place in high-income countries, did not include QI interventions, or did not include small and sick hospitalised newborns. Cochrane Risk of Bias tools were used to quality appraise the studies.

**Results:**

From 8110 results, 28 studies were included, covering 23 LMICs and 65,642 participants. Most interventions were meso level (district and clinic level); fewer were micro (patient-provider level) or macro (above district level). In-service training was the most common intervention subtype; service organisation and distribution of referencing materials were also frequently identified. The most commonly assessed outcome was mortality, followed by length of admission, sepsis rates, and infection rates. Key barriers to implementation of quality improvement initiatives included overburdened staff and lack of sufficient equipment.

**Conclusions:**

The frequency of meso level, single centre, and educational interventions suggests that these interventions may be easier for programme planners to implement. The success of some interventions in reducing morbidity and mortality rates suggests that QI approaches have a high potential for benefit to newborns. Going forward, there are opportunities to strengthen the focus of QI initiatives and to develop improved, larger-scale, collaborative research into implementation of quality improvement initiatives for this high-risk group.

**Trial registration:**

PROSPERO CRD42017055459.

**Electronic supplementary material:**

The online version of this article (10.1186/s13012-018-0712-2) contains supplementary material, which is available to authorized users.

## Background

Newborns, or infants under 28 days of age, account for the greatest proportion of deaths amongst under-five children. The vast majority of these deaths occur in low- and middle-income countries (LMICs), which in 2016 comprised 98.5% of the 2.61 million neonatal deaths worldwide [1]. The estimated average of neonatal mortality rates in LMICs was 20 per 1000 live births, compared to 3 per 1000 in high-income countries [1]. Targeting this high-risk group is thus an urgent policy priority, particularly regarding the three major causes of neonatal deaths, which are preterm birth complications, severe infections, and intrapartum complications [2].

Low birth weight newborns (birthweight of < 2500 g) are especially vulnerable. Newborns can have low birth weight due to prematurity and/or being small for gestational age (SGA). A neonatal mortality relative risk analysis found that preterm and SGA babies had relative risks of 6.82 and 1.83, respectively, compared to non-low birth weight infants [3]. Survivors of premature birth are at risk of cardiovascular dysfunction, chronic lung disease of prematurity, neurodevelopmental problems, and visual and sensory impairment [4]. SGA newborns likewise have a higher risk of stunting and later, cardiac, metabolic, and cognitive impairment (although to a lesser extent than preterm babies) [5, 6]. Babies who become ill also suffer severe consequences—meta-analysis of newborns in sub-Saharan Africa, South Asia, and Latin America estimated an incidence risk of 7.6% for possible severe bacterial infection, with an associated case-fatality risk of 9.8% [7]. Intrapartum complications can result in neonatal encephalopathy and longer term consequences of disability and cerebral palsy [8].

Most small and sick newborns require inpatient hospital care, ideally in a dedicated unit, and there is a great potential for quality improvement (QI) in this setting, especially in LMICs. Quality improvement is defined by Ovretveit as “better patient experience and outcomes achieved through changing provider behaviour and organisation through using a systematic change method and strategies” [9]. Change of behaviour and organisation can occur at different levels, for example, micro interventions, at the level of the patient-provider interaction (e.g., distribution of referencing materials to providers); meso interventions, at the district and clinic level (e.g., service organisation); or macro, above district level (e.g., regulation and governance) [10, 11]. The Lancet Every Newborn series estimated that increased coverage and improvements at and around the time of birth could avert 71% of deaths [12]. Preventing nosocomial infections [13, 14], irrational antibiotic usage [15], inadequate use of Kangaroo Mother Care [16], low adherence to use of breast milk [17], unsafe oxygen use [18], insufficient bonding with mothers and parents [19], and increasing adherence to humane [20] and baby friendly [21, 22] hospital care are all potential areas for QI. As LMICs are increasingly committing to plan and invest in hospital infrastructure and capacity building of health providers for small and sick newborn care, it is timely to strategise and support simultaneous quality improvement efforts.

Previous reviews that have touched on QI initiatives for newborns in LMICs have focused on maternal and child health outcomes together. Dettrick et al. found that evidence in this area is often poor, and most research focuses on service utilisation above other indicators [23]. Additionally, Althabe et al. conducted a review of systematic reviews [24]. Amongst reviews covering over 300 studies, only 18 of these were conducted in LMICs, illustrating the disparity in the amount of research undertaken in these countries [24]. Austin et al. examined approaches to improve maternal and newborn care across all types of countries and observed that quality improvement interventions in LMICs have tended to be community based, rather than hospital based [25].

This review seeks to contribute to the existing evidence base by synthesising data on quality improvement initiatives for hospitalised small and sick newborns in LMICs. We aim to address the following objectives within eligible studies:

- To identify and categorise quality improvement approaches for small/sick hospitalised newborns in LMICs
- To identify and categorise outcomes investigated by quality improvement initiatives for small/sick hospitalised newborns in LMICs
- To identify barriers and promoters, at a local level and systems level, to the implementation of quality improvement initiatives for small/sick hospitalised newborns in LMICs

Our work will serve as a guide to quality improvement initiatives in this area by synthesising evidence on approaches used, outcome measures employed to estimate effects, and the nature of implementation challenges, for the information of future healthcare workers undertaking similar initiatives which should be evidence-based.

## Methods

A protocol for this review was published on the PROSPERO register in January 2017, registration number CRD42017055459 (PROSPERO, 2017).

### Eligibility criteria

Studies were included if they met the following criteria:

- Populations: hospitalised small and/or sick newborns in LMICs and admitted for inpatient healthcare. LMICs were identified according to the World Bank list of LMICs [26]. Facilities for this population must be defined as ‘hospitals’ or units within hospitals.
- Interventions: quality improvement initiatives, according to the Ovretveit definition—“better patient experience and outcomes achieved through changing provider behaviour and organisation through using a systematic change method and strategies” [9].
- Outcomes: objective clinical outcomes relating to mortality, morbidity, and process of care measures.
- Language: studies published in English, or with translation available.
- Year: published from 2000 or later.

The year cut-off was chosen in order to focus the review on recent practice in the context of changing healthcare systems. Studies that focused solely or primarily on practices in the delivery room that encompassed small/sick newborns only as a subset, or studies that focused on community interventions, were deemed not to meet the eligibility criteria of *hospitalised* small and sick newborns. Outcomes of self-assessed competency, or patient-assessed satisfaction, were also deemed not to meet the eligibility criteria of clinical outcomes, as this method of assessment was seen to be an insufficient proxy for an objectively measured clinical outcome. Finally, in order to simplify the review, studies focusing only on implementation of Kangaroo Mother Care (KMC), a specific method of care for preterm infants that focuses on encouraging skin-to-skin contact between mother and infant, were not included unless the study involved modification to KMC implementation, because KMC has been well covered in systematic reviews elsewhere [27, 28].

### Information sources

We searched the following electronic databases from January 2000 onwards: Medline, EMBASE, WHO Global Health Library, and Cochrane Library. We searched the trial registries: WHO ICTRP and ClinicalTrials.gov for completed and ongoing studies. Searches were conducted in April 2017. The literature searches of peer-reviewed publications were supplemented by scanning the reference lists of relevant studies and systematic reviews. Interlibrary lending was used to access certain papers.

### Search strategy

The following search strategy was designed to capture studies that were suitable for inclusion in the review. For example, the search strings used in EMBASE were:

- Term 1: Quality improvement
  (Quality or performance or effectiveness) AND (care or improvement* or increase* or service$ or indicator$)
- Term 2: Newborns
  Neonat* or neo-nat* or Baby or Babies or Newborn$ or new-born$ or infant$
- Term 3: Hospitalised
  Inpatient$ or in-patient$ or hospitalis* or NICU or neonatal intensive care unit.
  These search terms were then combined to give a final search of Term 1 AND Term 2 AND Term 3, which was used to search abstracts in these databases.

### Study selection, extraction, and analysis

The titles and abstracts were screened by two researchers independently for inclusion/exclusion, with disagreements resolved with arbitration from a third reviewer. The results shortlisted for inclusion and then underwent full-text screening, again undertaken by two researchers independently with arbitration from a third, to produce a final shortlist of articles to be included in the review. Data were extracted from each paper by one researcher and checked by a second, using a piloted worksheet, the details of which are supplied in Additional file 1. Summary results from fields that are not presented in the main manuscript are available on request.

To standardise study classifications, the following definitions were used, based on NICE definitions [29]:

1. Randomised controlled trial—similar people are allocated, at random, to different groups in order to test the efficacy of an intervention, with one group receiving the tested intervention
2. Intervention study (non-random)—similar people are allocated, via a non-random process, to different groups in order to test the efficacy of an intervention, with one group receiving the tested intervention
3. Before and after study—dependent variables are assessed in a setting before and after an intervention is applied, where the population may be the same or differ

QI approaches were classified according to the Kruk and Gage schema ‘Synthesizing improvement approaches’ [10]. This classifies approaches at the micro, meso, or macro level, meaning at the level of the patient-provider interaction, such as on the sick newborn care unit; at the district and clinic level; at an individual hospital; or at the above district level, such as across a health system. It then provides sub-classifications of approaches within each of these classes.

The relevant outcomes of the studies were extracted. Each outcome was then classified by a research team member. These classifications were based on the WHO-defined components of ‘quality of care’ [30]. This states that high-quality care should be Safe, Effective, Timely, Efficient, Equitable, and People-centred. Studies were sub-sorted by results, according to whether they reported that there was a significant increase in the metric during their study, a significant decrease, no significant change, or if statistical significance was not assessed or not reported. Significance was defined as either *p* < 0.05 or using the 95% confidence interval.

Barriers and promoters of quality improvement were classified as local level, meaning individual to the particular hospital or location where the intervention was based, and systems level, meaning a factor that would necessarily influence hospitals and locations beyond the local area. We utilised a wide scope for extraction of barriers and promoters, including both barriers and promoters that were specific objects of study and those that were informally reported such as through staff feedback reported in the discussion.

In order to assess bias in the included studies, we utilised the Cochrane Risk of Bias for Non-Randomised Studies of Interventions (ROBINS-I) and revised tool for Risk of Bias in randomised trials (RoB 2.0) to assess included quantitative studies [31, 32]. Any study with a Critical ROBINS-I Overall Bias or a High RoB 2.0 Overall Bias classification was omitted from the “Results” and “Discussion” sections of this review. Two independent reviewers conducted the quality appraisal, and adjudication was provided by a third reviewer if warranted.

## Results

### Study selection

**Fig. 1:**
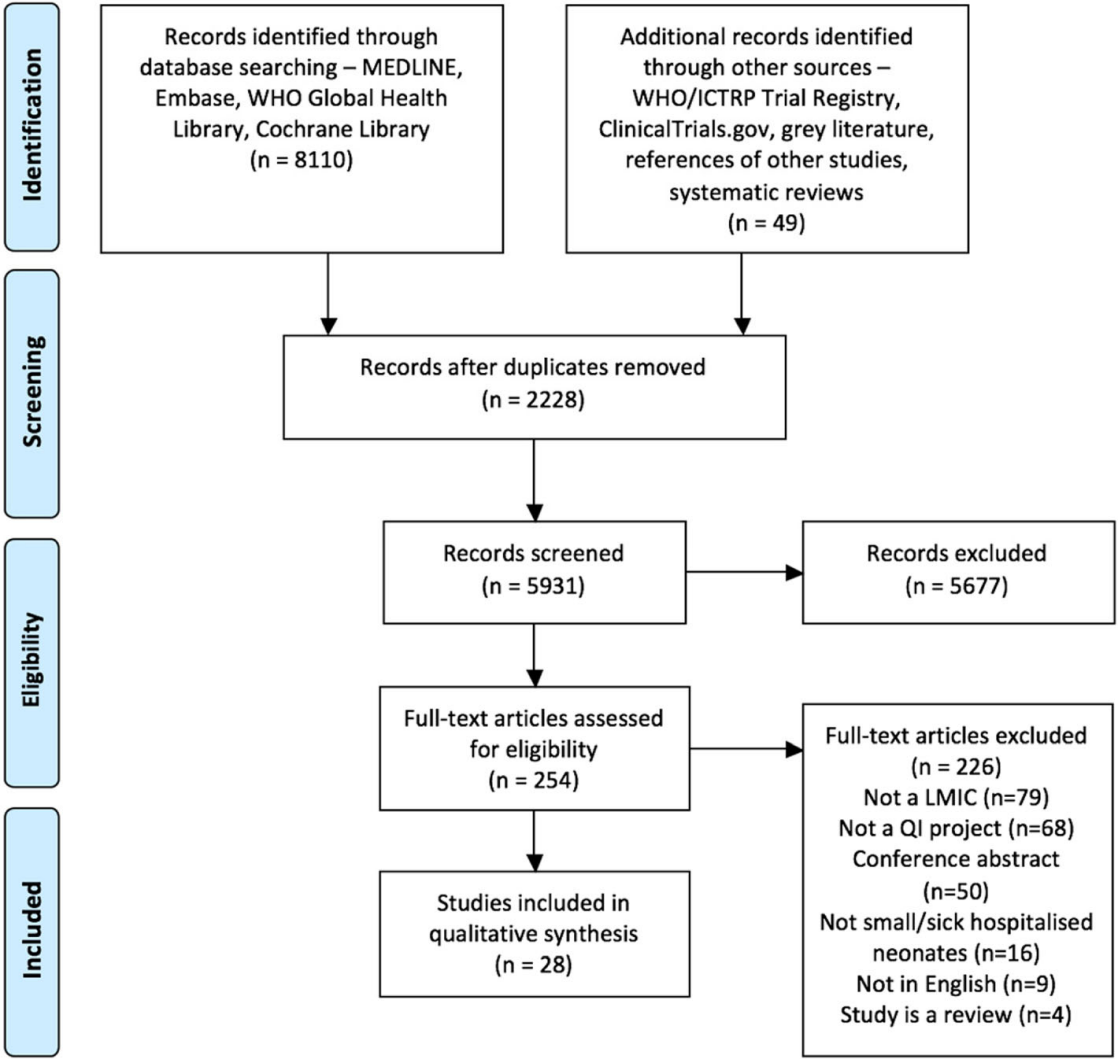
Screening strategy for included studies (PRISMA flow diagram) [75]

In total, the searches returned 8110 results across the four databases, with 49 results identified from other sources. After 2228 duplicates were excluded, a total of 5931 results’ titles and abstracts were screened against inclusion criteria. Of these, 5677 results were excluded for not meeting inclusion criteria at this stage, as demonstrated by Fig. 1.

This left 254 papers, which underwent full-text review. As shown in Fig. 1, the most common reason for exclusion at this stage was that studies took place in countries that were not LMICs (*n* = 79) according to the World Bank classifications [26]. The second most common reason was that studies were not describing QI interventions (*n* = 68). Twenty-eight studies were ultimately identified as suitable for inclusion in the narrative synthesis. Owing to the heterogeneity of QI methodologies, settings, and outcomes measured, meta-analysis was not undertaken.

### Study and participant characteristics

The 28 studies consisted of 25 controlled before and after studies, two intervention studies (non-random), and one randomised controlled trial. Table 1 provides a summary of all the included studies.

**Table 1:** Summary of included studies

Author	Study type	Location	Population	Sample size	QI measure	Outcomes
Agarwal et al. 2007 [36]	Controlled before and after study	India	Neonates born within the obstetric teaching hospital	15,249	Package of interventions including rational practice, protocol usage, training and empowerment of nurses	Mortality: 30% decline in NMR Length of admission: reduction from 8.6 days to 7.1. Sepsis: reduction in deaths due to sepsis from 37.9 to 15.5% Appropriate antibiotic use: antibiotics use decreased to 23.2%.
Bastani et al. 2015 [48]	Randomised controlled trial	Iran	Mothers with preterm infants	91	A family centred care (FCC) programme	Hospital admission/readmission: FCC group were significantly less likely to be rehospitalised, *p* = 0.04. Length of admission: 6.96 in FCC group, 12.96 in control group, *p* < 0.001. Maternal satisfaction: FCC group were significantly more satisfied.
Bhutta et al. 2004 [49]	Controlled before and after study	Pakistan	Very low birth weight infants	509	A step-down unit for mothers and babies	Mortality: rates of survival increased, from 65 to 84% (*p* < 0.05). Length of admission: length of stay fell from mean of 34 to 16. Patient weight gain: there was a reduction in mean weight at discharge from 1.6 to 1.289 kg (*p* < 0.001). Patient infection rates: rates of overall nosocomial infections dropped significantly. Sepsis: increased relative risk of culture proved neonatal sepsis (95% CI 0.92–1.26). Severe illness: non-significant changes in rates of intraventricular haemorrhage, apnoeic spells, respiratory distress, and necrotising enterocolitis; significant decrease in patent ductus arteriosus rates. Presence of hyperbilirubinaemia: rates fell from 28.8% to 17.9%.
Cavicchiolo et al. 2016 [58]	Controlled before and after study	Mozambique	NICU residents—inborn and outborn patients of all gestational ages up to the postnatal age of 7 days	4276	A continuous multi-level quality improvement intervention focused on infrastructure, equipment and protocol refinement	Mortality: reduction in death rate from 26 to 18%, significant. Hospital admission/readmission rate: admissions for prematurity, sepsis and asphyxia increased significantly. Sepsis: admissions for sepsis increased significantly, deaths decreased non-significantly. Severe illness: deaths for asphyxia increased significantly, admissions increased significantly.
Clark et al. 2012 [50]	Controlled before and after study	Sierra Leone	Children presenting for emergency care	500	Training course based on ETAT WHO course, ward combined to form ICU and ER, triage area created, improved equipment, experienced nurses in triage, structured clerking pack introduced	Mortality: decreased from 12.38 to 5.85%. Length of admission: no change.
Crouse et al. 2016 [38]	Controlled before and after study	Guatemala	Random sample of all patients presenting to the PED and all patients admitted to the PICU	1027	Emergency Triage Assessment and Treatment (ETAT)-based emergency triage process	Mortality: decreased from 12 to 6% amongst critically ill, not significant. Hospital admission/readmission: admission from the Paediatric Emergency Department fell significantly from 8 to 4%, and also fell significantly in critically ill group. Length of admission: decreased, not significant.
Darmstadt et al. 2005 [51]	Controlled before and after study	Bangladesh	Preterm infants in Special Care Nursery	–	Infection control programme	Mortality: decline in deaths of certain causes, significance not mentioned. Patient infection rates: decrease in nosocomial infection reports, and *K. pneumoniae*. Infection detection rates: decline in cases of culture-proven sepsis and suspected sepsis. Sepsis: significant decline in patients with clinical diagnosis of sepsis (79%). Appropriate antibiotic use: antibiotic use guidelines were reviewed, no data. Adherence to national guidelines of care: staff trained in standard guidelines, antibiotic guidelines were adhered to.
dos Santos et al. 2015. [47]	Intervention study (non-random)	Brazil	NICU newborns	24	NIPS scale; non-pharmacological actions in pain control in newborns	Adherence to national guidelines of care: significantly lower NIPS (pain scale) score with intervention.
Erdeve et al. 2008 [52]	Intervention study (non-random)	Turkey	All mother−preterm infant dyads that were consecutively admitted to the NICU	60	Use of individual rooms	Hospital admission/readmission: rehospitalisation rate was higher in non-intervention group *p* < 0.05. Length of admission: no significant difference regarding duration of intensive care hospitalisation. Patient weight gain: no significant change on discharge in body weight. Breastfeeding practice: no significant change in groups regarding breastfeeding rates.
Gathara et al. [39]	Controlled before and after study	Kenya	Sick newborns aged 0–7 days and malnourished children aged 6–59 months	798	Package of interventions including clinical guidance booklets, admission record form, a training course on emergency and admission care, external support supervision, local facilitation, performance assessment, and feedback	Mortality: mortality was reduced by 3% post intervention in intervention group, control group was static. Appropriate antibiotic use: overdoses of penicillin were reduced in intervention vs control group, but overdoses of gentamicin were increased. Adherence to national guidelines of care: documentation of gestation in weeks were increased in intervention group, and mean documentation score was higher. More vitamin K was prescribed in intervention groups.
Gilbert et al. 2014 [53]	Controlled before and after study	Brazil	Neonates admitted to NICU	1242	A 5-phase POINTS of Care package	Mortality: crude survival rates did not change over time significantly except in one NICU where it decreased. Patient weight gain: days to regain birth weight were significantly higher in post-intervention period. Retinopathy of prematurity: no significant change. Sepsis: rates did not change—11.3/12.3 cases per 1000 infant days. Lower respiratory tract disease: non-significant increase in bronchopulmonary dysplasia. Severe illness: non-significant increase in bronchopulmonary dysplasia, no change in necrotising enterocolitis.
Leng et al. 2016 [40]	Controlled before and after study	China	Very low birth weight neonates	172	Use of radiant warmers, warmer delivery room, STABLE programme, consulting services, standardised transportation, education of staff, review and feedback	Mortality: mortality rates decreased from 12 to 7%, *p* = 0.03. Length of admission: reduced from 60 to 45 days, *p* = 0.01 Sepsis: sepsis rates did not change significantly. Hypothermia rates: significant decrease in patients with temperatures < 36 degrees Celsius. Lower respiratory tract disease: percentage with chronic lung disease did not change significantly. Severe illness: rates of intraventricular haemorrhage and necrotising enterocolitis did not change significantly, but SNAPPE-II score increased significantly.
Mais et al. 2015 [41]	Controlled before and after study	Lebanon	Neonates with central lines in NICU	213	Theoretical and practical teaching sessions, dressing change guidelines, sterile technique, auditing adherence to guidelines	Length of admission: there was no significant change. Patient infection rates: CLABSI rates declined significantly, *p* < 0.05. Mechanical ventilation: no significant change. Central line duration: no significant decline in usage.
Namazzi et al. 2015 [42]	Controlled before and after study	Uganda	All pregnant and newly delivered mothers residing within the villages of the Iganga/Mayuge Health and Demographic Surveillance Site	–	District led training, support supervision, mentoring, supply of essential medicine and equipment	Mortality: hospitalised NMR declined from 17 to 9%, not significant. Kangaroo Mother Care: by the end of the study, 547 preterm babies had been cared for in a KMC unit. Premature delivery rate: rate was 8% in deliveries in health units.
Pinto et al. 2013 [43]	Controlled before and after study	Brazil	Newborns with very low birth weight	136	Dissemination of a new protocol proposed by the Brazilian National Health Surveillance Agency for antibiotic usage in LBW infants	Mortality: overall mortality decreased from 20.9 to 4.4%, significant. Patient infection rates: no significant change in multi-resistant infection rates. Sepsis: no difference in relation to confirmed sepsis, but a significant reduction in diagnoses of probable sepsis. Severe illness: no change in diagnoses of severe illnesses, e.g., PDA, PBD, necrotising enterocolitis. Appropriate antibiotic use: decrease in number of antimicrobial regimens used and days of antibiotic use.
Rahman et al. 2017 [44]	Controlled before and after study	Bangladesh	Children identified as having systemic sepsis	1036	Triage, fast assessment, immediate results, immediate antibiotics, training package, slow charts, checklist, records system, infection control measures, equipment stocking	Mortality: mortality decreased, significance not reported. Length of admission: increase in % with syndromic sepsis staying for over 48 h, significance not reported. Appropriate oxygen use: post intervention 94% were given oxygen with hypoxaemia. Appropriate antibiotic use: first-line recommended antibiotic usage increased from 49 to 75%, *p* < 0.005.
Ramaswamy et al. 2015 [59]	Controlled before and after study	Ghana	Obstetric and neonatal cases in regional referral facilities	–	Ridge-Kybele model for obstetric and neonatal care—an integrated approach to systems change	Adherence to national guidelines of care: 37% improvement in NICU hand hygiene rates. Waiting times: 74% reduction in mothers with unacceptable waiting times.
Rosenthal et al. 2012 [33]	Controlled before and after study	Argentina, Colombia, El Salvador, India, Mexico, Morocco, Peru, the Philippines, Tunisia, Turkey	NICU patients	6829	VAP (ventilator-associated pneumonia) bundle—11 items	Patient infection rates: ventilator-associated pneumonia rates per 1000 mechanical ventilator days decreased from 17.8 to 12.0. Lower respiratory tract disease: ventilator-associated pneumonia rates per 1000 mechanical ventilator days decreased from 17.8 to 12.0. Adherence to National Guidelines of Care: hand hygiene compliance rates rose from 62 to 81%. Mechanical ventilation: days of MV did not change.
Rosenthal et al. 2013 [34]	Controlled before and after study	El Salvador, Mexico, Philippines, and Tunisia	NICU patients with central line insertion	2214	INICC multidimensional infection control approach	Patient infection rates: CLABSI rate reduction from baseline of 54%, 95% CI 0.33–0.63 RR. Adherence to National Guidelines of Care: hand hygiene and sterile gauze rates rose significantly.
Salehi et al. 2015 [45]	Controlled before and after study	Iran	Hospitalised ‘infants’	100	Implementation of guidelines and education	Patient weight gain: patients in intervention group had a mean weight change of + 96 g compared to − 59, *p* = 0.001.
Sethi et al. 2017 [54]	Controlled before and after study	India	Preterm neonates	26 neonates, 23 mothers	CPNC—comprehensive post-natal counselling package, comprising education of health care providers and family members	Breastfeeding practice: the proportion of mothers expressing milk on day 1 increased to 86.6% from 12.5%, after 1 year the proportion of neonates on exclusive breast milk was more than 80%.
Soni et al. 2016 [55]	Controlled before and after study	India	Infants admitted to a rural Indian neonatal intensive care unit (NICU)	648	Presence of physician champions	Length of admission: length of stay was greater with champions, at 9 days, compared to 7 without, *p* = 0.01. Patient infection rates: patients who experienced infections decreased significantly as physician champions left. Appropriate antibiotic use: no association between champions and antibiotic usage. Breastfeeding practice: breastfeeding rates were not changed. Usage of Kangaroo Mother Care: skin to skin care increased with champions and lasted longer hours per day. Premature delivery rate: with KMC champions there was a higher percentage of premature deliveries, *p* = 0.01 for trend.
Srofenyoh et al. 2012 [35]	Controlled before and after study	Ghana	Mothers and neonates in Ridge Regional Hospital	29,508	An interdisciplinary approach, high-level sponsorship, establishment of guidelines, measurement, feedback, leadership and teamwork coaching, training including QI training, and a multimodal focus on patients, providers, and systems	Mortality: perinatal mortality was reduced, no information on significance. Maternal satisfaction: this improved. Maternal health: 34% decrease in maternal mortality. Stillbirth: reduced by 36%, *p* < 0.05.
UNICEF 2014 [37]	Controlled before and after study	Bangladesh	Hospitalised newborns	–	Quality improvement initiatives delivered alongside SCANUs—Special Care Newborn Units	Mortality: average case fatality rates dropped in most SCANUs. Hospital admission/readmission: admissions at SCANUs increased.
Wrammert et al. 2017 [56]	Controlled before and after study	Nepal	Neonates in maternity hospital, Kathmandu	299	Implementation of Helping Babies Breathe Protocol	Mortality: decrease in death rate in first 24 h, *p* < 0.01. No significant change in 7/28 day mortality.
Yawson et al. 2016 [60]	Controlled before and after study	Ghana	Users of Ghanaian newborn care service	–	BNA tool to identify service gaps with group discussions, leading to national and regional operational plans and monitoring/evaluation framework	Mortality: mortality reduced in the intervention regions.
Zhou et al. 2013 [57]	Controlled before and after study	China	All neonates who received mechanical ventilation for at least 48 h and were hospitalised in the NICU for ≥ 5 days	491	A bundle of comprehensive preventive measures against VAP were gradually implemented using the evidence-based practice for improving quality method.	Mortality: mortality rates decreased from 14% in phase 1 to 3% in phases 2 and 3, statistically significant. Patient infection rates: sustained decline in VAP rates, *p* = 0.01.
Zhou et al. 2015 [46]	Controlled before and after study	China	Neonates in the NICU	171	EPIQ programme—team taught for 2 days, who then identified strategies for adoption of CLABSI prevention, and trained other members	Patient infection rates: CLABSI rates declined in each successive phase. Central line duration: time in situ increased across the phases, significance not reported.

The 28 included studies included data from 23 different countries. This included data from India (4 studies), China (3 studies), Bangladesh (3 studies), Ghana (3 studies), Brazil (3 studies), Iran (2 studies), El Salvador (2 studies), Mexico (2 studies), the Philippines (2 studies), Tunisia (2 studies), Turkey (2 studies), Uganda, Nepal, Kenya, Guatemala, Mozambique, Lebanon, Sierra Leone, Pakistan, Argentina, Colombia, Peru, and Morocco. The review includes two multi-country analyses, with one covering Argentina, Colombia, El Salvador, India, Mexico, Morocco, Peru, the Philippines, Tunisia, and Turkey and the other covering El Salvador, Mexico, the Philippines, and Tunisia [33, 34].

There were a total of 65,642 reported participants included in analyses across the studies, although one large study included 29,508 deliveries in its analysis and another included 15,249 [35, 36].

Table 2 presents the ROBINS-I and RoB 2.0 ratings awarded to each of the studies. One study scored Critical in Overall Risk of Bias, and thus was excluded from data synthesis, but is included in Tables 1 and 2 [37].

**Table 2:** Quality appraisal of included studies

ROBINS-I tool for non-randomised studies of interventions								
Studies	Bias due to confounding	Bias in selection of participants into the study	Bias in classification of interventions	Bias due to derivations from intended interventions	Bias due to missing data	Bias in measurement of outcomes	Bias in selection of the reported result	Overall bias
Agarwal et al. [36]	Moderate	Low	Low	Low	Low	Low	Moderate	Moderate
Bhutta et al. [49]	Moderate	Low	Low	Low	Low	Moderate	Serious	Serious
Cavicchiolo et al. [58]	Moderate	Low	Low	Low	Low	Moderate	Moderate	Moderate
Clark et al. [50]	Serious	Low	Low	Low	Low	Low	Moderate	Serious
Crouse et al. [38]	Moderate	Low	Low	Low	Serious	Low	Moderate	Serious
Darmstadt et al. [51]	Moderate	Low	Low	Low	No info	Low	Moderate	Moderate
Dos Santos et al. [47]	Serious	NI	Serious	Low	Low	Serious	Moderate	Serious
Erdeve et al. [52]	Moderate	Low	Low	Low	Low	Moderate	Moderate	Moderate
Gathara et al. [39]	Moderate	Moderate	Low	Low	Serious	Moderate	Moderate	Serious
Gilbert et al. [53]	Moderate	Low	Low	Low	Moderate	Low	Moderate	Moderate
Leng et al. [40]	Moderate	Low	Low	Low	Low	Moderate	Moderate	Moderate
Mais et al. [41]	Moderate	Low	Low	Low	Low	Low	Moderate	Moderate
Namazzi et al. [42]	Serious	Low	Low	Low	No info	Moderate	Moderate	Serious
Pinto et al. [43]	Moderate	Low	Low	Low	Low	Low	Moderate	Moderate
Rahman et al. [44]	Moderate	Low	Low	Low	No info	No info	Moderate	Moderate
Ramaswamy et al. [59]	No info	No info	No info	No info	No info	No info	Serious	Serious
Rosenthal et al. [33]	Moderate	Low	Low	Low	Low	Moderate	Moderate	Moderate
Rosenthal et al. [34]	Moderate	Low	Low	Low	Low	Low	Moderate	Moderate
Salehi et al. [45]	Serious	Low	Low	Low	No info	Low	Low	Serious
Sethi et al. [54]	Serious	Low	Low	Low	Low	Low	Moderate	Serious
Soni et al. [55]	Moderate	Low	Low	Low	Serious	Low	Moderate	Serious
Srofenyoh et al. [35]	Moderate	Low	Low	Low	Low	Low	Moderate	Moderate
UNICEF [37]	No info	No info	No info	No info	No info	No info	Critical	Critical
Wrammert et al. [56]	Moderate	Low	Low	Low	Low	Low	Moderate	Moderate
Yawson et al. [60]	No info	No info	Low	Low	No info	No info	Serious	Serious
Zhou et al. [57]	Serious	Low	Low	Low	Low	Low	Moderate	Serious
Zhou et al. [46]	Serious	Low	Low	Low	Low	Low	Moderate	Serious
Risk of Bias 2.0 tool for randomised studies								
Studies	Bias arising from the randomisation process	Bias due to deviations from intended interventions	Bias due to missing outcome data	Bias in the measurement of the outcome	Bias in the selection of the reported result	Overall bias		
Bastani et al. [48]	Low	Medium	Low	Medium	Low	Medium		

The core narrative themes extracted from the papers are presented under the headings, classified QI approaches, groups of outcomes measured in QI approaches, and barriers and promoters to implementing QI approaches. Henceforth, summary data comes from the 28 studies with Overall Risk of Bias of Serious or lower.

### Classified quality improvement approaches

We categorised the overarching approaches for quality improvement used for sick newborn care using the Kruk and Gage ‘Synthesizing improvement approaches’ schema [10]. We found 11 studies with micro interventions [33, 38–47], 23 studies with meso interventions [34–36, 38–42, 44–46, 48–59], and two studies with macro interventions [59, 60]. Nine of the studies had mixed-level interventions, with eight of these being meso and micro and one being macro and meso.

Table 3 provides information on approaches for quality improvement by subtype. In addition to the included subtypes in Table 3, there were additional categories according to the Kruk and Gage schema, for which no studies utilised those particular methods; these were, at the macro level, pay for performance, other financing and incentives, pre-service training, and external to health system and, at the meso level, mortality audits and social franchising [10].

**Table 3:** Subtype of intervention

Level	Strategy	Total	Citation
Micro	Distribution of referencing materials to providers	8 studies	[33, 38, 39, 41, 43–46]
	Decision support	2 studies	[39, 40]
	Care coordination	5 studies	[33, 39, 42, 46, 47]
Meso	Strengthening facility infrastructure	6 studies	[35, 42, 44, 50, 57, 58]
	Continuous quality improvement	7 studies	[34, 35, 41, 46, 51, 58, 59]
	Supervision	5 studies	[35, 39, 51, 55, 57]
	Feedback	6 studies	[34, 35, 39, 40, 51, 59]
	In-service training	20 studies	[34–36, 38–42, 44–46, 48, 50, 51, 53, 54, 56–59]
	Service organisation	9 studies	[35, 36, 39, 40, 44, 49, 50, 52, 59]
Macro	Regulation and governance	1 study	[59]
	Task shifting	1 study	[60]

The most frequent subtype of intervention was the meso approach ‘In-service training’, used by 20 studies. Such interventions were often delivered as part of a group of innovations—for example, Rosenthal et al. introduced a multifaceted infection control bundle incorporating education on hand hygiene and asepsis, and Agarwal et al. developed a package of interventions that included on-job training of nurses in common neonatal skills [34, 36]. The intervention of Clark et al. was based on a WHO Emergency Triage and Treatment training course, and Sethi et al. utilised a Comprehensive Post-Natal Counselling package, comprised of education of health care providers and family members [50, 54]. In Zhou et al., key staff members attended a training workshop run by the Canadian Neonatal Network for 2 days, and attendees in turn then trained the other Neonatal Intensive Care Unit (NICU) team members [46].

The second most frequent subtype of intervention was the meso approach, ‘Service organisation’, used by nine studies. For example, Rahman et al. described an approach that involved service reorganisation with triage and fast assessment and use of a record system [44]. Erdeve et al. evaluated the impact of individual rooms on patients and families in the NICU [52].

The most frequent micro approach was ‘Distribution of Referencing Materials to Providers’ by eight studies. Pinto et al. disseminated a new protocol proposed by the Brazilian National Health Surveillance Agency for antibiotic usage in low birth weight infants [43]. Salehi et al. also described the implementation of new guidelines, as did Mais et al. and Gathara et al. as part of their approaches [39, 41, 45].

Two studies utilised macro approaches, which were regulation and governance, and task shifting. Ramaswamy et al. used regulation and governance in their development of the Ridge-Kybele Model for Obstetric and Neonatal Care, an integrated approach for systems change which prioritises capacity building in order to properly embed change practices [59]. Yawson et al. utilised task shifting, by using a tool to identify service gaps which led to national and regional operating plans being developed and implemented to improve neonatal care [60].

### Groups of outcomes measured in quality improvement approaches

A total of 23 broad outcomes were used to assess the efficacy of the QI interventions, shown in Table 4. The majority of these, 13, were outcomes that were classified as aiming at delivering safe care, defined as “delivering health care which minimises risks and harm to service users, including avoiding preventable injuries and reducing medical errors” [30]. Five were aimed at delivering effective care, two efficient care, two people-centred care, and one timely care. No studies were identified as including outcomes addressing the delivery of equitable care.

**Table 4:** Quality improvement outcomes

Quality of care classification of QI outcome measure	Quality improvement outcome	Significant increase	Significant decrease	No significant change	Significance not assessed or not reported
Safe (minimising risks and harm)	Mortality	–	8 studies—[36, 40, 43, 49, 50, 56–58]	4 studies—[38, 42, 53, 56]	5 studies—[35, 39, 44, 51, 60]
	Patient weight gain	1 study—[45]	2 studies—[49, 53]	1 study—[52]	–
	Patient infection rates	1 study—[55]	7 studies—[33, 34, 41, 46, 49, 51, 57]	1 study—[43]	–
	Effect on retinopathy of prematurity	–	–	1 study—[53]	–
	Sepsis rates	–	3 studies—[36, 43, 51]	5 studies—[40, 43, 49, 53, 58]	–
	Rates of hypothermia	–	1 study—[40]	–	–
	Patient lower respiratory tract disease	–	1 study—[33]	2 studies—[40, 53]	–
	Severe illness (various)	2 studies—[40, 58]	1 study—[49]	4 studies—[40, 43, 49, 53]	–
	Presence of hyperbilirubinaemia	–	–	1 study—[49]	–
	Effect on breastfeeding practice	1 study—[54]	–	2 studies—[52, 55]	–
	Maternal health	–	1 study—[35]	–	–
	Stillbirth	–	1 study—[35]		
	Premature delivery rate	1 study—[55]	–	–	1 study—[42]
Effective (utilising evidence)	Appropriate oxygen use	–	–	–	1 study—[44]
	Antibiotic usage	1 study—[44]	2 studies—[36, 43]	1 study—[55]	2 studies—[39, 51]
	Adherence to national guidelines of care	4 studies—[33, 34, 47, 59]	–	–	2 studies—[39, 51]
	Mechanical ventilator days	–	–	2 studies—[33, 41]	–
	Central line duration	–	–	1 study—[41]	1 study—[46]
Efficient (avoiding waste)	Length of admission	1 study—[55]	4 studies—[36, 40, 48, 49]	2 studies—[38, 41]	3 studies—[44, 50, 52]
	Hospital admission/readmission	1 study—[58]	3 studies—[38, 48, 52]	–	1 study—[49]
People-centred (accounting for preferences of service users)	Usage of Kangaroo Mother Care	1 study—[55]	–	–	1 study—[42]
	Maternal satisfaction	1 study—[48]	–	–	1 study—[35]
Timely (reducing delays)	Waiting times	–	–	–	1 study—[59]

### Mortality rate

Mortality rate was the most frequently measured outcome, assessed by 16 studies. Studies used a variety of mortality metrics, including 28-day mortality, mortality within the study period, and specific cause mortality. Eight studies found that introduction of the QI intervention was associated with a significant decrease in mortality, four found no significant change, and five others reported results but not the statistical significance of those results. Amongst the more successful interventions, Bhutta et al. found that survival in their NICU increased from 65 to 84%, *p* < 0.05, after a policy change to create a stepdown unit and involve mothers earlier in the care of their at risk infants [49]. Pinto et al. also found a significant reduction in mortality, from 20.9 to 4.4%, *p* = 0.009, after the dissemination of a new antibiotic protocol to their NICU, which was supervised by two neonatologists in charge of the clinical routine [43]. Leng et al. also found mortality rates decreased, from 12 to 7%, *p* = 0.03, amongst newborns transferred from eight hospitals to their Level III NICU, after the introduction of a package of interventions including the STABLE programme and staff education [40]. Crouse et al. found that overall mortality for the critically ill patients decreased with their new emergency triage process, from 12.4% pre-intervention to 6.0% post-intervention, but this was not statistically significant (*p* = 0.15) [38].

### Length of admission

Length of admission was the second most assessed outcome, by ten studies. Studies reported mixed outcomes, with one reporting a significant increase, four a significant decrease, two no change and three where significance was not assessed. Soni et al. found that length of stay in the NICU increased with identified Kangaroo Mother Care champions, at a median of 9 days, compared to a median of seven without the champions [61]. Conversely, Bastani et al. found that, in their RCT, mean length of stay in the NICU for their family-centred care group was 6.96 days compared to 12.96 in the control group, *p* < 0.001 [48]. Bhutta et al. found that length of stay more than halved after their stepdown unit was introduced, from a mean of 34 days pre-intervention to 16 post-intervention [49].

### Sepsis rates

Sepsis rates were the joint third most frequently measured outcome, assessed by eight studies. Three studies reported a significant decrease in sepsis rates, and five reported no significant change. Amongst the studies that reported significant decreases were Agarwal et al., who found that there was a severe reduction in deaths in their neonatal unit due to sepsis after the introduction of their multi-faceted intervention package, from 37.9% pre-intervention to 15.5% post, *p* < 0.01 [36]. Their package included greater involvement of mothers in caregiving, as with Bhutta et al., alongside enforced aseptic routines, greater use of protocols, education, and other features [36, 49]. However, in Gilbert et al., the introduction of an educational package, POINTS of Care, did not change sepsis rates across five neonatal units nor did the multi-faceted intervention incorporating education, feedback, and other elements in Leng et al. [40, 53].

### Patient infection rates

Patient infection rates, across a variety of different specific infections, were assessed by nine studies. Seven studies reported a significant decrease in infection rates, one a significant increase and one reported no significant change. Amongst the studies that reported significant decreases was Rosenthal et al., who found that a multi-faceted ventilator-associated pneumonia (VAP) bundle intervention was associated with a reduction in VAP rates from 17.8/1000 ventilation days pre-intervention to 12.0/1000 ventilation days post-intervention, across 15 NICUs in ten countries [33]. Rosenthal et al. found that after an infection control bundle, central line-associated bloodstream infection (CLABSI) rates reduced across four NICUs in four countries, resulting in a relative risk of 0.45 post-intervention (95% CI 0.33–0.63) [34]. Both interventions utilised infection control teams and surveillance. Mais et al. also reported on CLABSI rates, which declined from 15/1000 central line days before the introduction of a bundle of interventions to 6.4/1000 afterwards (*p* < 0.05) [41]. For Zhou et al., CLABSI rates also fell significantly from 16.7/1000 before the introduction of a nursing training programme to 5.2/1000 afterwards [46].

### Barriers and promoters to implementing quality improvement approaches

Many factors have the potential to either promote or inhibit the successful implementation of interventions for quality improvement [62, 63], and several of these were covered by the studies. These factors are noted in Table 5 and are identified as either barriers or promoters operating at a local or systems level. In total, 11 barriers were identified, with six at the local level and five at the systems level, and 13 promoters were identified, with nine at the local level and four at the systems level. No study assessed the statistical significance of any barrier or promoter; identified barriers and promoters were largely inductive by the investigators.

**Table 5:** Factors influencing efficacy of QI measures

Local level	Studies	Systems level	Studies
Promoters			
Motivation of key individuals	3 studies—[35, 42, 51]	Relationships between health workers, community leaders and district officials	1 study—[42]
Continuous monitoring throughout	2 studies—[38, 56]	High-quality national data collection	1 study—[60]
Interdisciplinary collaboration	2 studies—[35, 38]	Formal health service support	1 study—[35]
Abandonment of unnecessary practices	1 study—[36]	NGO collaboration initiatives	1 study—[58]
Schemes tailored to participants	1 study—[38]		
On-site support	1 study—[44]		
Refresher programmes	1 study—[44]		
Formal training in QI methods	1 study—[35]		
Low cost of intervention	1 study—[38]		
Barriers			
Overburdened staff	4 studies—[36, 42, 53, 56]	Insufficient funding	1 study—[42]
Lack of sufficient equipment	4 studies—[36, 38, 42, 58]	Insufficient health services relative to demand	1 study—[42]
High changeover of workforce	3 studies—[35, 36, 53]	Government redistribution of staff	1 study—[53]
Defects in staff knowledge and practice	1 study—[35]	Inadequate documentation	1 study—[39]
Unmotivated staff	1 study—[53]	Confounding health policy changes	1 study—[50]
Multiple QI measures/audits simultaneously	1 study—[55]		

### Promoters

Nine promoters were identified at the local level by seven studies. Three studies highlighted the importance of motivation of key individuals. Darmstadt et al. highlighted the will and effort of key individuals as being important, especially the nursing supervisor; Srofenyoh et al. and Namazzi et al. noted the importance of local champions [35, 42, 51].

Four promoters were identified at the systems level by four studies. Yawson et al. stated that good quality national data collection is essential for designing QI interventions [60]. Namazzi et al. highlighted structured community relationships, Cavicchiolo et al. said NGO collaboration could be helpful, and Srofenyoh et al. valued formal support from the Ghanian Health Service [35, 42, 58].

### Barriers

Six barriers to quality improvement initiatives were identified at a local level by eight studies. The barrier posed by overburdened staff was the joint most frequently mentioned barrier, by four studies. This includes Gilbert et al. who mentioned staff being overstretched as an impediment, and Namazzi et al. who discussed the fact that facility staff had competing demands on their time [42, 53]. Four studies also mentioned the problem of insufficient equipment, such as Crouse et al. who stated that paper supplies running low and no computerised patient records hampered record keeping [38].

Five barriers were identified at a systems level by four studies. Namazzi et al. highlighted both the problems of increasing demand for services at all hospitals and lack of finances for necessary medicines [42]. Gilbert et al. said government policies enforcing redistribution of staff from study NICUs to underserved areas also created barriers [53].

## Discussion

Many LMICs have focused on developing the infrastructure for inpatient care of sick newborns in public health systems. The recent focus on quality of care with the launch of the Quality, Equity and Dignity Network co-led by the WHO and United Nations International Children’s Emergency Fund (UNICEF), alongside ongoing efforts to raise the standard of paediatric care, has increased the interest in addressing gaps in quality of care for sick newborns. To our knowledge, this is the first systematic review to specifically examine quality improvement initiatives for hospitalised small and sick newborns in LMICs. Previous reviews have focused on maternal and child care as a whole [23, 24], or did not focus on LMICS individually [25], or have focused on a subtype of quality improvement interventions (in service training) [64], with all of these also encompassing non-hospitalised newborns.

### Programmatic implications

Programme planners should consider that the majority of interventions are at the meso level, and many studies (20 studies) involved in-service training as part of their interventions. Educational interventions may be frequently represented because exposure of the intervention to the relevant staff can be appropriately monitored, implemented, and tailored to local needs, whereas macro-level interventions like regulation and governance or task shifting require greater continuous coordination. For planners designing their first QI projects, in-service training may be an advisable first step. However, structural adjustments remain necessary in order to facilitate QI at all levels of care. It will be especially relevant to formulate and implement policies to retain skilled nursing staff alongside providing financing to achieve national standards of minimum infrastructure and equipment, and to increase staffing for optimal nurse-patient ratios. Increasing adoption of perinatal death reviews in LMICs will also provide an opportunity to review and address common gaps in a country through macro-level interventions.

The most frequently assessed outcomes were mortality rates, sepsis and infection rates. Eight of the 12 studies that investigated statistically significant differences in mortality rates observed a statistically significant decrease in mortality. Particularly high reductions occurred in Bhutta et al. and Pinto et al., with mortality falling by 19% in the former after the creation of a stepdown unit and mortality falling by 16.5% in the latter after the dissemination of a new antibiotic protocol [43, 49]. These studies demonstrate the potential for QI to produce swift and significant benefits for this vulnerable patient group. However, it is important to note that the majority of the included studies were non-randomised before-after studies, and a review by Schouten et al. found that observational studies tend to demonstrate larger effects than more rigorous designs [65]. There are many feeders into mortality rates and a number of the studies had important confounders. With regards to other outcomes, in general, studies did not report greater involvement of family members as part of their interventions, but those that did reported significant positive results for mortality, sepsis, and readmission rates, suggesting this area could be explored further [36, 48, 49]. It will be important to focus future QI efforts on sepsis due to rising rates of antibiotic resistant infections and sepsis in this group [66]. Some successful studies in this area did incorporate holistic interventions involving family members [36, 49].

Several studies benefitted from focusing their efforts on single pathologies (ventilator-associated pneumonia, central line-associated blood stream infections) for quality improvement, which allowed for collation of findings across multiple centres [33, 34]. Other interventions utilised training courses included the POINTS of Care training (a six module training programme covering topics including pain control and nutrition interventions) [53, 67] and the STABLE programme (a continuing education course that focuses on stabilising sick newborns) [40]. Such approaches allow easy monitoring of attendance and hence exposure for future studies that may choose to utilise control groups.

For programme planners, our identification of barriers and promoters to successful QI interventions will be helpful to accelerate efforts for meeting the objectives of Every Newborn Action Plan, Every Woman Every Child, and other quality of care agendas [68, 69]. Planners should consider resolving barriers such as overburdened staff, which may be more severe during times of higher seasonal demand, and consider identifying ‘staff champions’ to promote their projects which were reported as promoters in several studies [35, 42, 51]. A previous review of barriers and enablers of KMC identified similar influential factors, such as the barrier posed by a high workload [70].

Finally, it is important to reflect that quality improvement approaches are heavily dependent on data linked to action and evidence to drive positive change. Moxon et al. have advocated for the urgent need to improve health management information systems and monitoring of hospital care for newborns for better measurement of quality of care and to identify and address quality gaps [62]. Thus, the allocation of resources on improving quality of sick newborn care needs to factor in requirements for improving local, country-led sustainable information systems, as well as systematic use of perinatal death audits [71]. Mortality audits were not utilised by any of the included studies, despite a recent focus via Maternal and Perinatal Deaths Surveillance and Response projects [72]. The bottlenecks of health financing, health workforce, data and community engagement need more varied approaches to implementation and research, especially at Macro level, and advanced information systems would be valuable in order to optimise QI if used for action at the correct level of the health system.

### Research implications

Regarding future research into QI initiatives in LMICs, we recommend QI implementation takes place in tandem with strong data collection and monitoring. The majority of research takes place in high-income countries, whose settings may not be fully applicable to LMICs [12, 25]. We also note the impact of several interventions was hard to assess because studies did not analyse statistical significance. These interventions would benefit from further, larger-scale studies, or more rigorous evaluation. The sharing of outcome measures across studies would also allow for improved future quantitative synthesis.

We found that research often focused on single centres, also noted by Dettrick et al. [23]. More work should be done to evaluate quality improvement at multiple centres in different settings, such as comparisons between rural and urban hospitals, and to investigate the impact of scaling up existing projects [12, 73]. This may need specific capacity building of public health programmes and clinical staff on research in LMICs and provision of grants to undertake context-specific projects on a range of interventions. Barriers and promoters were not assessed for statistical significance by any study, and multi-centre studies could enable such analysis and the provision of richer qualitative data. Generally, we would recommend that QI projects are rigorously evaluated and the experience documented or reported in peer-reviewed literature wherever feasible.

### Limitations of our study

The heterogeneity of our study population, the interventions for quality improvement and multiple outcome measures were key challenges. Quality improvement approaches is an umbrella term for a variety of interventions, and though we cast a wider net, some studies may have been missed from our selection criteria, especially if they did not use the exact term. Many healthcare interventions in LMICs are community- rather than hospital-based, and care during pregnancy was not considered, so many interventions that may be useful in mitigating overall newborn morbidity and mortality were not eligible [74]. In general, many quality improvement projects do not progress to published literature, particularly small-scale projects, so publication bias is likely to be present. Unpublished ongoing initiatives ongoing in collaboration with WHO, UNICEF, USAID and others could not be included.

This study did not capture solely qualitative literature, and qualitative synthesis would be useful going forward, particularly for identifying barriers and promoters. There was generally little information on the methodology for identifying barriers and promoters in the included studies, which may mean that findings are not fully representative. The study method did not allow for collection of information relating to parental experience, which is a critical dimension of quality of care improvement. This study is also limited by only including studies published from 2000 onwards and published in English. We did not conduct overall outcome-specific assessments of quality of evidence with a tool such as GRADE. Finally, although we aimed to analyse with reference to wealth, rural/urban, and type of facility as measures of healthcare equity, data on financing of health settings were available for only a minority of the included papers, and many hospital settings were anonymised. As a result, these analyses were not undertaken.

## Conclusion

Going forward, we recommend more rigorous evaluation of quality improvement in neonatal hospital care. Interventions are commonly at the meso level and educational in nature, and more focus is required around macro- and micro-level interventions; other study designs should be explored, with direct investigation of barriers and promoters. This should be linked to programmatic efforts where possible, in order to combine implementation and research. Small and sick hospitalised newborns in LMICs are a population at the highest risk—they should be one of the prime beneficiaries of quality of care interventions and investments. Targeted resources will be needed to strengthen human resource capabilities for implementation research into quality improvement for small and sick newborn care and to document outcomes, costs, and lessons learnt.

## Additional file

Additional file 1: Pilot extraction worksheet. List of fields which were extracted from studies where available. (DOCX 109 kb)

## Abbreviations

CLABSI: Central line-associated bloodstream infections
ICTRP: International Clinical Trials Registry Platform
KMC: Kangaroo Mother Care
LMICs: Low- and middle-income countries
NICU: Neonatal Intensive Care Unit
POINTS of Care: Educational package for control of pain, oxygenation, infection, nutrition, and temperature and to improve supportive care
QI: Quality improvement
SGA: Small for gestational age
STABLE: Sugar, temperature, airway, blood pressure, lab work, and emotional support
UNICEF: United Nations International Children’s Emergency Fund
USAID: United States Agency for International Development
VAP: Ventilator-associated pneumonia
WHO: World Health Organization
