# Supplementary material for: Quality improvement initiatives for hospitalised small and sick newborns in low- and middle-income countries: a systematic review
Source: Implement Sci. 2018 Jan 25;13:20. doi: 10.1186/s13012-018-0712-2 (PMC5784730; doi:10.1186/s13012-018-0712-2)
Supplement: Additional file 1: — Pilot extraction worksheet. List of fields which were extracted from studies where available. (DOCX 109 kb) [file 13012_2018_712_MOESM1_ESM.docx]

**Piloted extraction worksheet**

The piloted extraction worksheet included the following fields: Country, Specific location in country, Study type, Study objectives, Study year, Publication Year, Population, Sample Size, Children age range, Children gender distribution, Wealth of population, Details of ‘sickness’ of children, Details of prematurity of children, Details of secondary co-morbidities of children, Sampling and Recruitment Methods, Religion, Ethnicity (incl. generation), Eligible, Recruited, Lost to follow up, Analysed, Data Collection Methods, Key quality improvement intervention, Classification (regulatory/organisational/educational/clinical), Type of health facility, Financing of health setting, Primary outcome measure, secondary outcome measure, Effect of QI project on 7 day mortality rates, Effect of QI project on 28 day mortality rates, Effect of QI project on mortality rates (general), Effect of QI project on hospital admission/readmission, Effect of QI project on average length of hospital stay, Effect of QI project on patient weight gain, Effect of QI project on patient head circumference, Effect of QI project on patient length, Effect of QI project on patient infection rates, Effect of QI project on patient infection detection rates, Effect of QI project on retinopathy of prematurity, Effect of QI project on sepsis, Effect of QI project on hypothermia rates, Effect of QI project on lower respiratory tract diseases, Effect of QI project on severe illness, Effect of QI project on Kangaroo mother care, Effect of QI project on appropriate oxygen use, Effect of QI project on appropriate antibiotic use, Effect of QI project on adherence to national guidelines of care, Effect of QI project on presence of hyperbilirubinaemia, Effect of QI project on breastfeeding practice, Effect of QI project on maternal satisfaction, Effect of QI project on maternal health, Effect of QI project on maternal-infant attachment, Effect of QI project on fetal distress, Effect of QI project on premature delivery rates, Effect of QI project on neonatal hypoglycaemia, Effect of QI project on macrosomia, Effect of QI project on other outcome measures, Identified barriers to successful quality improvement, Identified promoters of successful quality improvement, Other comments to aid interventional development.
